# Rapid and sensitive detection of ovarian cancer biomarker using a portable single peak Raman detection method

**DOI:** 10.1038/s41598-022-13859-x

**Published:** 2022-07-21

**Authors:** Mohesh Moothanchery, Jayakumar Perumal, Aniza Puteri Mahyuddin, Gurpreet Singh, Mahesh Choolani, Malini Olivo

**Affiliations:** 1 Translational Biophotonics Laboratory, Institute of Bioengineering and Bioimaging, Agency for Science, Technology and Research (A*STAR), Singapore, Singapore; 2 Department of Obstetrics and Gynecology, Yong Loo Lin School of Medicine, National University of Singapore, Singapore, Singapore; 3 Present Address: Centre for Preclinical Imaging, Faculty of Health and Life Sciences, University of Liverpool, Liverpool, UK

**Keywords:** Biochemistry, Cancer, Biomarkers, Diseases, Oncology, Engineering, Materials science, Optics and photonics

## Abstract

Raman spectroscopy (RS) is a widely used non-destructive technique for biosensing applications because of its ability to detect unique ‘fingerprint’ spectra of biomolecules from the vibrational bands. To detect these weak fingerprint spectra, a complex detection system consisting of expensive detectors and optical components are needed. As a result, surface enhanced Raman spectroscopy (SERS) method were used to increase the Raman signal multifold beyond 10^12^ times. However, complexity of the entire Raman detection system can be greatly reduced if a short wavelength region/unique single spectral band can distinctly identify the investigating analyte, thereby reducing the need of multiple optical components to capture the entire frequency range of Raman spectra. Here we propose the development of a rapid, single peak Raman technique for the detection of epithelial ovarian cancers (EOC)s through haptoglobin (Hp), a prognostic biomarker. Hp concentration in ovarian cyst fluid (OCF) can be detected and quantified using Raman spectroscopy-based in vitro diagnostic assay. The uniqueness of the Raman assay is that, only in the presence of the analyte Hp, the assay reagent undergoes a biochemical reaction that results in product formation. The unique Raman signature of the assay output falls within the wavenumber region 1500–1700 cm^−1^ and can be detected using our single peak Raman system. The diagnostic performance of our Raman system had 100.0% sensitivity, 85.0% specificity, 100.0% negative predictive value and 84.2% positive predictive value when compared to gold standard paraffin histology in a proof-of-concept study on 36 clinical OCF samples. When compared to blood-based serum cancer antigen 125 (CA125) levels, the Raman system-based assay had higher diagnostic accuracy when compared to CA125, especially in early-stage EOCs.

## Introduction

Raman spectroscopy provides a “fingerprint” spectrum of narrow peaks, representing a specific set of biomolecules from the inelastic scattering of photons by molecules^1,2^. The majority of the photons interacting with molecules are elastically scattered, also known as Rayleigh scattering, while only a small portion (10^–10^ times of the incident photons) will be absorbed and re-emitted with a frequency shift due to the molecular vibrational modes^3^. This inelastic scattering of the photons is referred to as Raman scattering, which can be captured by a spectrometer to form the Raman spectra. Sensitive spectrometers with advanced optics are required to detect and enhance these weak Raman signals. Alternatively, Raman signals may be enhanced by using surface enhanced Raman spectroscopy (SERS) and by utilizing molecules adsorbed onto nano-roughened metal surfaces or onto colloidal metal nanoparticles^4,5^.

Commercial Raman systems with cooled CCD camera are fitted with advanced optical and laser components, therefore making the systems costly and bulky. However, if the quantitative measurement of the investigating analyte can be made from a specific spectral band (where significant change is present), measurement and processing of the entire Raman spectrum using a sophisticated high-end system is not required. The complexity and cost of the measurement setup can be reduced greatly if the focus is on a selected sensitive spectral band. The development of an affordable and portable Raman system with high detection sensitivity will enable smoother adoption of the technology for clinical translation. Herein, we describe the development of an ultrasensitive portable single peak Raman reader for the detection and quantification of ovarian cancer biomarker.

Ovarian cancer is the most common gynaecological cancer and in almost 90% of cases, it arises from the epithelial tissue layer covering the ovaries, termed epithelial ovarian cancer (EOC)^6^. Five-year prognosis can be > 90% if detected in the early stage and will be < 40% if detected at a later stage^7,8^. The delay in diagnosis for women at an early stage of EOC is mainly because of its vague symptoms and asymptomatic nature. Early detection and treatment will decrease mortality from this disease. However, no clinically proven and effective screening method currently exists and routine screening for asymptomatic ovarian cancer is not recommended^9^. CA125 blood test is widely used for ovarian cancer screening but the levels can also be elevated in benign conditions such as endometriosis and fibrosis, making it an unreliable diagnostic method^10^. Paraffin section histopathology is the "gold standard" for diagnosing ovarian cancer. It may take up to two weeks to get the results and it may involve a second operation for the returning patients. Early diagnosis and appropriate primary surgery could improve the survival rate in ovarian cancer. Intraoperative diagnosis is crucial in determining the choice and extent of surgical procedures, especially in young patients to preserve fertility^11^, and in patients undergoing laparoscopic surgery for ovarian pathology^12^. Even though frozen section (FS) is the only intraoperative diagnostic tool widely used to screen ovarian cancer, it is not commonly available in most parts of developing countries and in addition it suffers from accuracy variations and misdiagnosis due to large cyst size, non-uniform tissue malignancy, and limited staining methods^13^.

Haptoglobin (Hp) is an acute phase serum glycoprotein, which is present in human serum in small quantities. An elevated Hp concentration observed in the patient's serum and especially in the fluid present within the ovarian cyst, can be related to ovarian cancer^14^ . Usual methods for the detection of Hp include enzyme-linked immunosorbent assay (ELISA), time-resolved immune fluorometry, electrochemical impedance spectroscopy, chemiluminescent imaging and chromogen staining which all are tedious and time-consuming procedures^15–18^. UV absorbance is another widely available method for the detection of Hp, but it results in poor sensitivity at lower concentrations due to turbidity or blood contamination from the cyst fluid samples^14^. Hence, there is a need for a diagnostic solution which is sensitive to detect lower concentrations of Hp rapidly and not affected by any sample contamination. We have previously demonstrated the detection of Hp from OCF using surface enhanced Raman spectroscopy (SERS)-based assay and a commercial Raman Microscope which is not affected by any contamination^19,20^. However, the commercial Raman system used was bulky, expensive, and not easily translatable for clinical applications. Moreover, an ideal SERS substrate should have high SERS enhancement, be affordable, good stability and high reproducibility with long shelf life. This is still not fully addressed in existing SERS substrates^21^.

To demonstrate the versatility and sensitivity of our miniaturised portable Raman system, we have done the rapid diagnosis of epithelial ovarian cancer. The enzymatic assay for Hp detection was previously reported^19,20^. Briefly, Hp complex catalyzes 3, 3’, 5, 5’-tetramethylbenzidine (TMB) substrate to TMB^2+^ product. TMB is Raman inactive whereas the TMB^2+^ state is strongly Raman active. The Raman peak produced is within the wavelength region of 1500–1700 cm^−1^ and is the region of interest. Therefore, we propose the detection and quantification of this single peak Raman signal directly from OCF to differentiate between benign and malignant tumors. The ability to quantify Hp biomarker from the OCF within minutes has the potential for intraoperative diagnosis of ovarian cancer.

## Methods

### System design

**Figure 1:**
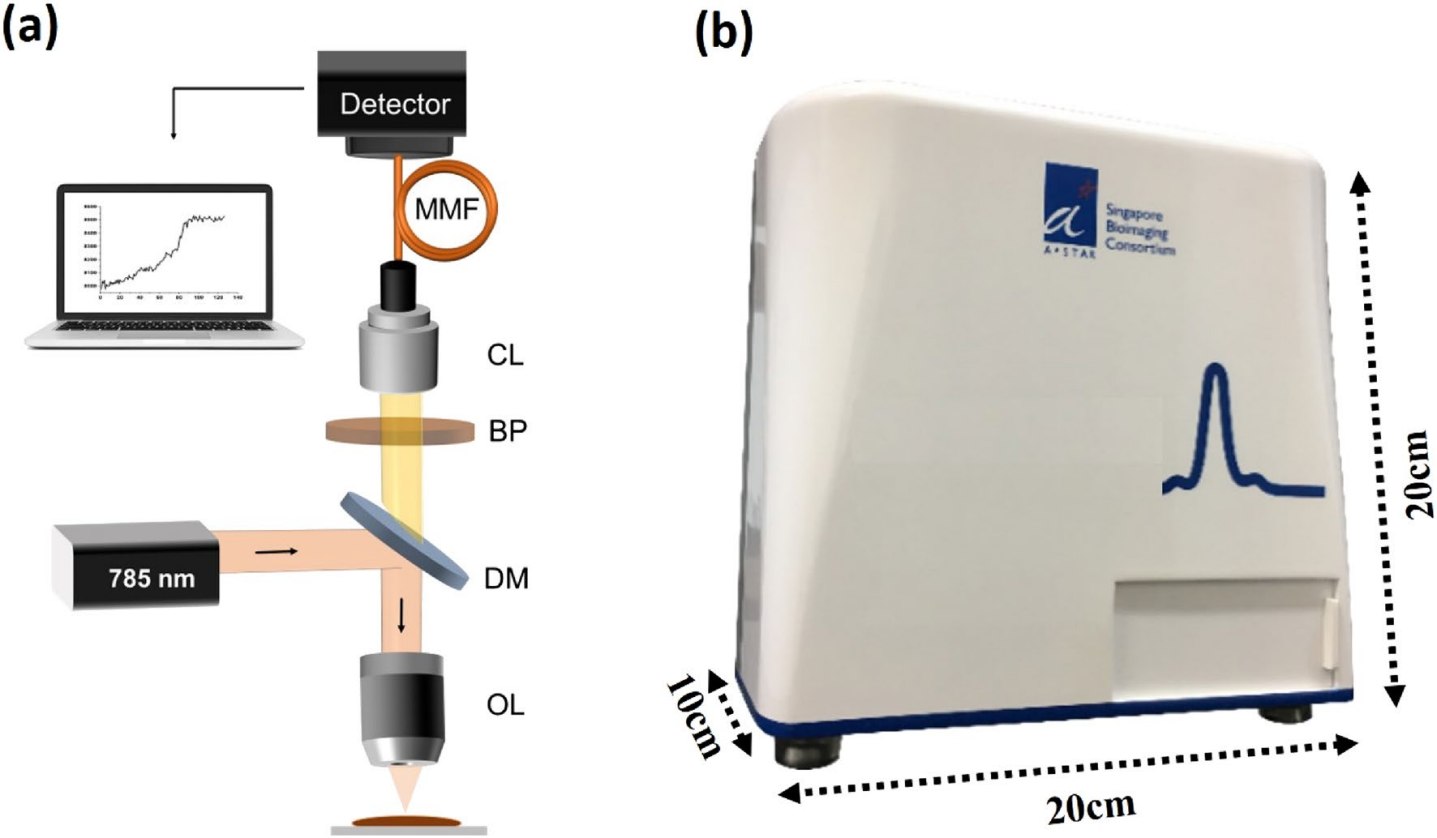
(**a**) Schematic of the single peak Raman reader. *OL* objective lens, *DM* dichroic mirror, *BP* band pass filter, *CL* collection lens, *MMF* multimode fiber. (**b**) Laboratory prototype of the single peak Raman reader.

The system was designed as shown in Fig. 1. Briefly, the 785 nm laser beam was deflected by a dichroic mirror (DM) onto the objective lens (OL), which focus the laser beam onto the sample. The intensity after the objective was 20 mW. The Raman signals from the sample were passed through the same OL to the collection lens (CL). Detection of the single peak of interest and the elimination of all other wavelength bands was achieved by placing a band-pass filter (BP) in front of the collection collimator. The single peak intensity was detected directly by a Complementary Metal Oxide Semiconductor (CMOS) sensor through a multimode fiber (MMF). The integration time of the camera was 1 s. Figure 1b shows the alpha prototype of the single peak Raman reader. The total cost of the current system is ~ 7.5 K USD, which can be further reduced using low-cost diode lasers and components.

### Diagnostic assay preparation

All the chemicals were purchased from Sigma-Aldrich (St. Louis, MO) such as 3, 3’, 5, 5’-tetramethylbenzidine and citrate buffer solution. Similarly, all the biologicals were purchased from abcam (Cambridge, MA). namely hemoglobin [Hb], Haptoglobin [Hp] of human origin. Hp forms an irreversible complex with Hb in a ratio of [1: 0.5–0.9]. Purified Hp (ab90924) or clinical OCFs were mixed with fixed concentration of Hb in equal volumes and incubated for approximately 10 min to form [Hb-Hp] complex. TMB reagent was added to [Hb-Hp] complex and allowed to react at room temperature for few mins. At the end of the reaction time stop solution was added to the mixture to quench the reaction. 10–20 µL of the reaction mixture was added onto a glass slide with a microwell and measured using the single peak Raman reader.

### Informed consent and guidelines

All methods were carried out in accordance with relevant guidelines and regulations. For the clinical sample collection, Ethics and governance approvals were obtained from local human research ethics committee National Health Group (NHG) Domain Specific Review Board (DSRB). Clinical samples were collected after obtaining written informed consent from each subject in accordance with the local ethics committee approved DSRB protocol (Protocol no: 2000/00856 and 2007/240).

### Clinical sample collection

Briefly, OCF samples were aspirated from the ovarian cyst without any spillage into the abdomen during surgery. The cyst fluid was centrifuged at 2000×*g* for 10 min at 4 °C.The supernatant was collected and stored at –80 °C until analysis. On the day of analysis, the OCF samples could come to room temperature prior to testing.

A total of 36 retrospectively archived OCF samples (16 malignant and 20 benign) were tested in this study. Of the 16 malignant samples, eight samples were stage I (50.0%), three stage II (19.0%), three stage III (19.0%), and two stage IV (12.5%) (Table 1). All ovarian cancer samples were staged and defined by histopathologic diagnoses according to the International Federation of Gynaecology and Obstetrics Committee^22^.

**Table 1:** Clinical characteristics of patients from whom ovarian cyst fluid samples were collected.

Cancer stage	Benign (n = 20)	Malignant (n = 16)
I	NA	8
II	NA	3
III	NA	3
IV	NA	2

## Results and discussion

### Single peak Raman detection of Hp

The single peak Raman reader was designed to detect a specific spectral band where significant change is present. For our study’s purpose, the reader was able to detect the Raman signal between the wavenumber regions 1550–1650 cm^−1^. A standard curve or calibration plot was performed using the single peak reader to known concentrations of Hp standard (0.42, 0.83, 1.65 and 3.30 mg/mL). The results from the single peak reader were compared to the data from a commercial Raman system. The averaged (*n* = 5) Raman intensity was obtained using a commercial Raman Microscope (InVia; Renishaw) for each Hp standard after processing the Raw spectra. Similarly, an averaged (*n* = 5) Raman intensity was obtained using single peak reader for the same Hp standards. All the clinical samples were measured in solution form (we drop 10–20 µL of mixed sample onto a glass slide with barrier for Raman measurement). To eliminate any background signals, we have subtracted the background of the Raman signals with 0.5% BSA solution in a similar glass slide before starting the experiments. Hence, we make sure the signal originating from the clinical sample will be only from the TMB^2+^ product in presence of Hp complex.

**Figure 2:**
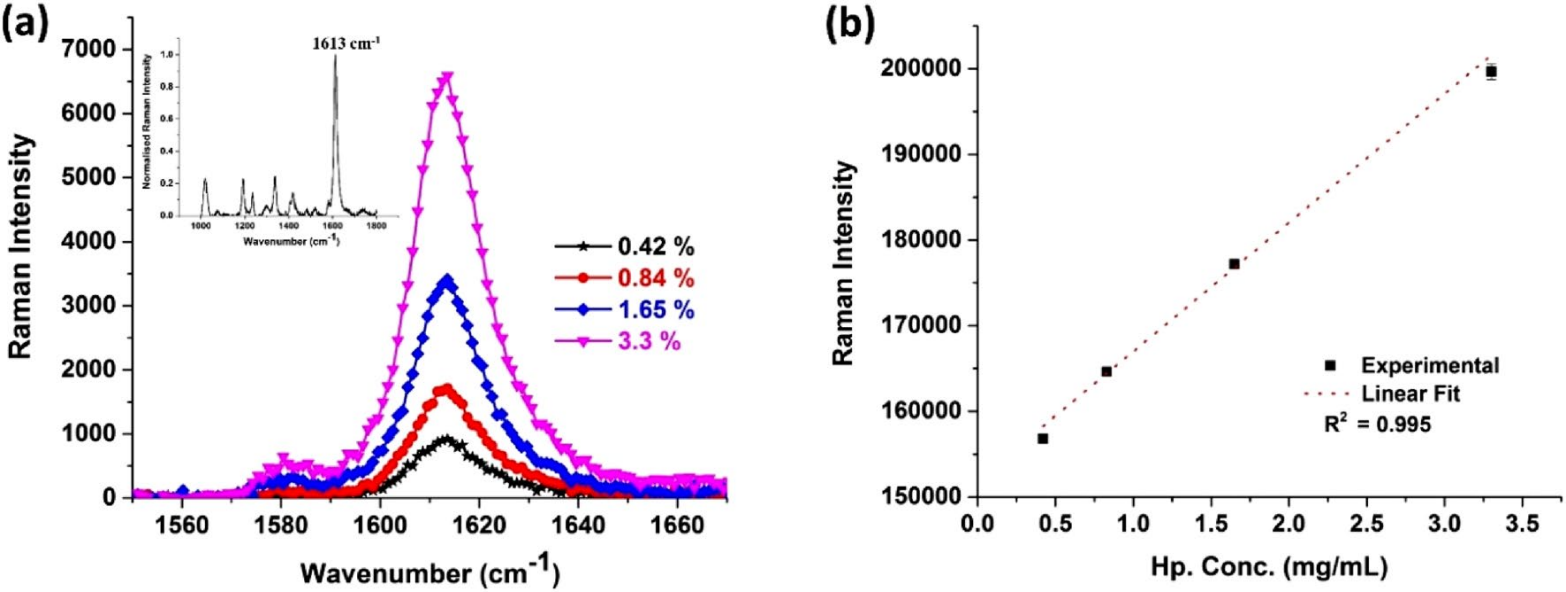
Calibration plots for the Raman assay obtained using (**a**) commercial Raman microscope: inlet showing the entire Raman spectra with the most prominent peak at 1613 cm^−1^; (**b**) prototype single peak Raman reader.

Figure 2a shows the averaged single peak between the wavenumber region 1550–1670 cm^−1^, detected using a commercial Raman microscope with a 785 nm excitation and a 50× objective lens. The system uses a spectrograph with 1,800 lines/mm grating and a cooled charge-coupled device (–70 °C) whereas the insert shows the entire Raman spectrum with peaks at 1019, 1192, 1236, 1339, 1419, 1613 cm^−1^. Among the various peaks 1613 cm^−1^ is very distinct and well suited for the quantification process to indirectly quantify Hp concentration present in the analyte sample. It is not easy to quantify varying concentration of hp from other mentioned peaks, because of the weak signals and expected variability. Therefore, we designed the prototype single peak Raman reader to quantify the representative peak at 1613 cm^−1^. The averaged Raman intensity was plotted against the known concentrations (in mg/ml) of Hp using our prototype single peak Raman reader and fitted into a linear curve (Fig. 2b). We obtained good linear correlation with an R^2^ value of 0.995 using our portable single peak reader with similar correlation (R^2^ = 0.997) from that of a commercial Raman microscope. The method combining Raman assay and single peak Raman reader will be known as “Raman system” from this point onwards.

### Diagnostic performance of Hp detection in OCF based on Raman vs histology

The diagnostic performance of Hp detection using Raman system was verified against histology on 36 OCFs (Table 2) using VassarStats: Statistical Computation Web Site. The Raman system was able to differentiate benign cyst and malignant tumours with 100% sensitivity (95% CI 76–100%) and 85% specificity (95% CI 61–96%). This resulted in a positive predictive value [PPV] of 84.2% (*n* = 17/20) for benign samples and a negative predictive value [NPV] of 100% (*n* = 16/16) for malignant samples.

**Table 2:** Diagnostic performance of Raman vs histology: sensitivity, specificity, PPV, and NPV.

Performance test	Raman disease		
	CA	Non-CA	Total
Positive	16	3	19
Negative	0	17	17
Total	16	20	36

	Estimated value (%)	95% confidence interval	
		Lower (%)	Upper (%)
Sensitivity	100	76	100
Specificity	85	61	96
PPV	84.2	59.5	96
NPV	100	77.1	100

*CA* cancer, *non-CA* non-cancer, *PPV* positive predictive value, *NPV* negative predictive value.

**Figure 3:**
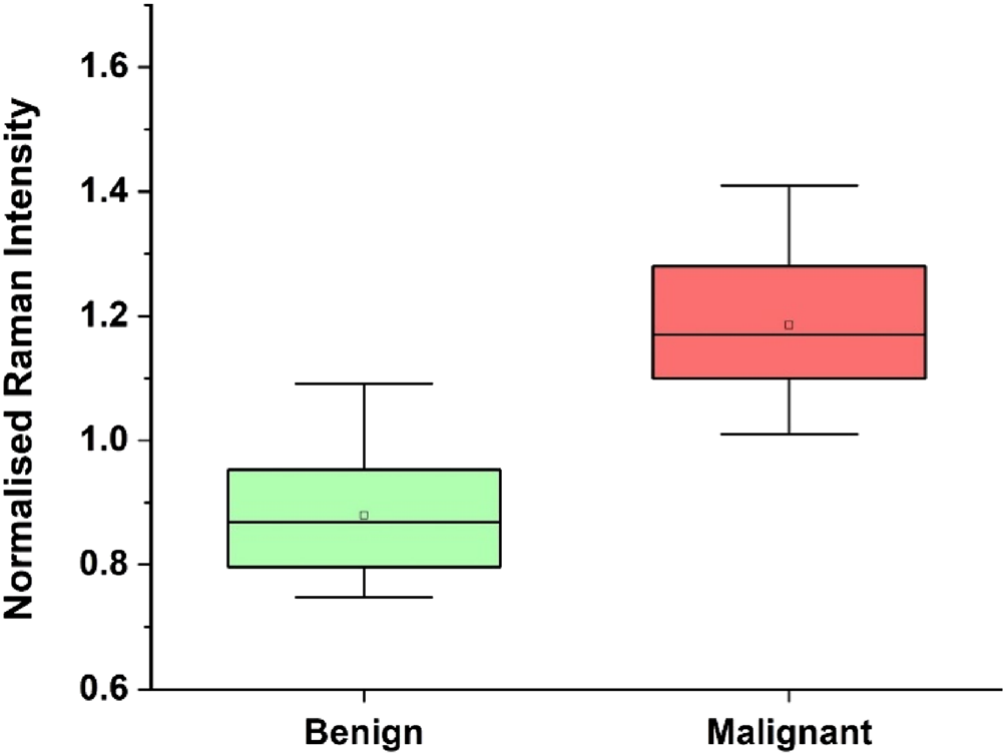
Classification of benign and malignant samples based on the Raman intensity.

**Figure 4:**
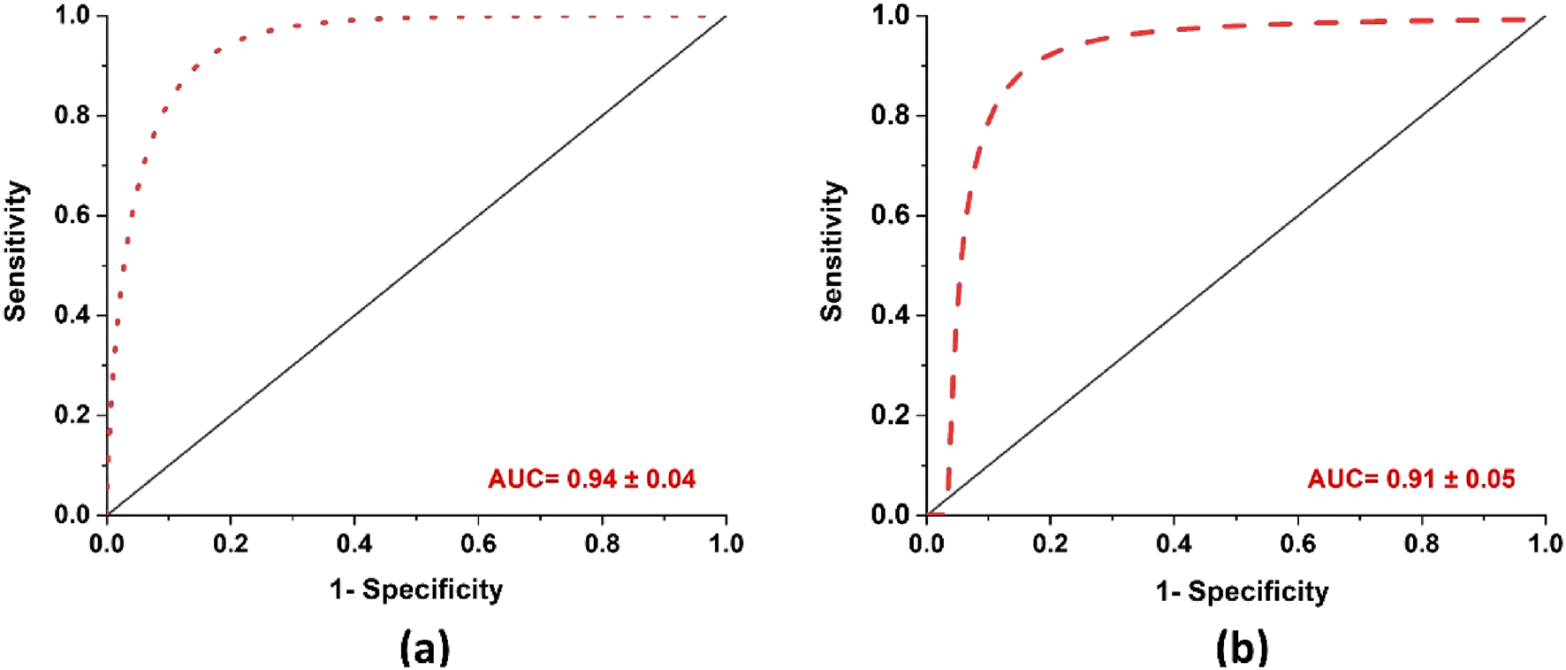
Fitted ROC curve using nonlinear least square model. (**a**) Raman system: mean AUC be 0.94 ± 0.04, (**b**) CA125: mean AUC be 0.91 ± 0.05.

Figure 3 shows the box plot of benign and malignant samples based on the normalized Raman intensity from the Hp within the OCF samples. From the figure there is marked variation in the Hp level present in Benign and Malignant OCFs, the Hp level is substantially high for Malignant compared to that of Benign OCFs. Figure 4 shows the receiver operating characteristic (ROC) curves, in which true positive rate (sensitivity) were fitted against false-positive rate (1 − specificity) using nonlinear least square model. Comparison of the mean area under the curve (AUC) and standard error from ROC between the Raman system and CA125, showed that the Raman system performed marginally better than CA125, 0.94 ± 0.04 and 0.91 ± 0.05 respectively, Fig. 4a,b.

### Diagnostic performance of CA125 against Raman detection of Hp

Clinical serum CA125 test with a cut-off of 35 U/mL has been used to screen for ovarian cancer risk. We compared the performances of serum CA125 on all 36 patient samples (using VassarStats: Statistical Computation Web Site) verified against histology (Table 3). Serum CA125 was able to differentiate benign and malignant tumors with 87.5% sensitivity (95% CI = 60.4–98%) and 90% specificity (95% CI = 67.0–98.2%). The method gave a PPV, of 87.5% (*n* = 14/16) for benign samples and NPV of 90% (*n* = 18/20) for malignant samples. Whereas using the Raman system to detect Hp, we correctly identified 100.0% of malignant samples (n = 16/16) and 85.0% of benign samples (n = 17/20), thus Raman detection technique of Hp had similar or superior diagnostic accuracy than serum CA125. CA125 had 2 False Negative (FN) and 2 False positive (FP) vs Raman system has no FN and 3 FP (Table 4).

**Table 3:** Diagnostic performance of CA125 vs histology: sensitivity, specificity, PPV, and NPV.

Performance test	CA125 disease		
	CA	Non-CA	Total
Positive	14	2	16
Negative	2	18	20
Total	16	20	36

	Estimated value (%)	95% confidence interval	
		Lower (%)	Upper (%)
Sensitivity	87.5	60. 4	98.0
Specificity	90.0	67.0	98.2
PPV	87.5	60.4	98.0
NPV	90.0	67.0	98.2

*CA* cancer, non-CA non-cancer, *PPV* positive predictive value, *NPV* negative predictive value.

**Table 4:** Diagnostic performance of CA125 and Raman vs histology on malignant samples.

S-ID	ST	GR	HP	CA125	CA125-DG	R	R-DG
86	1	1	TP	145.9	TP	1.28	TP
247	1	2	TP	113.0	TP	1.31	TP
1184	1	3	TP	2609	TP	1.12	TP
1190	3	3	TP	964.2	TP	1.01	TP
1483	1	3	TP	120.5	TP	1.17	TP
1484	2	3	TP	37.9	TP	1.11	TP
1754	4	3	TP	2060.0	TP	1.39	TP
1782	2	2	TP	567.3	TP	1.08	TP
1208	4	3	TP	5398.0	TP	1.19	TP
1221	1	1	TP	375.9	TP	1.33	TP
1261	3	2	TP	211.8	TP	1.07	TP
1286	1		TP	886.3	TP	1.04	TP
1321	1	2	TP	25.7	FN	1.22	TP
1333	3	3	TP	368.2	TP	1.20	TP
1382	1	4	TP	18.3	FN	1.41	TP
1410	2	3	TP	850.9	TP	1.11	TP

*S-ID* sample ID, *ST* stage, *GR* grade, *HP* histopathology, *R* Raman, *TN* true negative, *DG* diagnostics, *FN* false negative, *FP* false positive.

### Diagnostic performance of serum CA125 and Raman vs histology

We compared the performance of the Raman system and serum CA125 against the gold standard paraffin histopathology results (Table 3) on all malignant samples. Serum CA125 was able to differentiate malignant tumors with 87.5% sensitivity. Whereas Raman system correctly identified 100% of malignant samples (*n* = 16/16) thus Raman detection technique of Hp had superior diagnostic accuracy compared to CA125. Of the 16 malignant samples, two samples were reported as benign (false negative) in the CA125 test whereas the Raman system made the correct diagnosis (true positive). Therefore, the sensitivity and NPV for the Raman system is superior when compared to CA125 for malignant tumors. Whereas of the 20 benign samples, two samples were reported as malignant (false positive) in the CA125 test and using the Raman system three samples were reported as malignant (false positive). This is mainly due to the selection of cut-off value of Hp to do the normalization, in the case of Raman system.

We have successfully developed a Raman system i.e. an assay coupled with a single peak Raman reader prototype, to detect and quantify the presence of Hp, a prognostic biomarker for EOC. We showed that the Raman system was able to differentiate benign from malignant ovarian tumors with high diagnostic performance. Using the single peak Raman reader, we obtained good linear correlation (R^2^) value when compared to a commercial Raman microscope. The Raman system are designed to be used as an intraoperative diagnosis setup for detecting ovarian cancer.

The usefulness and clinical relevance of our miniaturized portable Raman system was demonstrated by its ability for rapid diagnosis of epithelial ovarian cancer. In this study, we did not utilize enhancing mediums such as plasmonic nanoparticle or nanofabricated sub-nanometer metal structures for the enhancement of intrinsic weak Raman signals as previously described^19^. Instead, Hp biomarker in OCF is quantified by means of the intensity of the pure Raman signal of TMB^2+^. The main advantage of this Raman system is that for biomarkers where the detection can be made from a single peak, this setup will be highly favourable, as the cost of systems development is significantly reduced.

Many patients undergoing surgery may end up losing complete ovaries due to absence of any conclusive inter-operative diagnostics. This is often due to the complicated decision-making procedures for the surgeon in the case of premenopausal women who require a fertility-sparing surgery. Single peak Raman detection of Hp can be used as a diagnostic tool to make quick clinical decisions regarding malignancy of the tumor during surgery.

Most ovarian cancers are asymptomatic at early stages. The current diagnosis of ovarian cancer is based on menopausal status, CA125 blood test and ultrasonography, which forms the Risk of Malignancy Index (RMI). This indexing method works well for postmenopausal women and those with advanced stage ovarian cancer. CA125 levels may also be elevated in other benign conditions such as endometriosis and fibrosis, hence may cause false positives especially in benign gynaecological conditions. In this study, the CA125 test was able to identify 87.5% of malignant samples (*n* = 14/16, 2 FN) and 90.0% of benign samples (*n* = 18/20, 2 FP). Intraoperative FS has been proposed as an alternative; however, the lengthy process involved in processing the tissue and the wide range of misdiagnosis rates, FS remains an evaluation tool and not diagnostic. A limitation of our study is the unavailability of FS results for most of the clinical OCF samples, therefore, we did not include FS in our comparative study. While paraffin histological examination of the tumour samples is the current gold standard, the turn-around-time for this technique is 1–2 weeks. An intraoperative diagnostic test must deliver rapid results whilst able to differentiate between benign and malignant cysts with an acceptable level of diagnostic accuracy. Malignant OCF representing cancers from all stages were included in the study to gain better understanding of Raman system diagnostic performance. Raman system had superior diagnostic accuracy compared to CA125 in identifying tumour malignancy especially in early-stage cancers (Stage 1/2), as shown in Table 3. Additionally, the current limitations of the small sample size require further validation involving larger clinical cohorts to assess the potential utility of this Raman system in clinical settings.

## Conclusion

In conclusion, the importance and usefulness of a portable and affordable Raman reader for the rapid detection of Hp to differentiate between benign and malignant tumors are reported. With increasing concentrations of Hb–Hp complex, a linear increase in the single peak Raman signal was monitored and quantified using a cutom made single peak Raman reader. Based on this, the concentration dependent peak of Hp at 1613 cm^−1^ was quantified in clinical samples. The performances of Hp detection based on a single peak Raman reader were compared to CA125 test against gold standard paraffin histopathology results on 36 patient samples. CA125 test had a sensitivity of 87.5% and specificity of 90.0% whereas Raman system had a sensitivity of 100.0% and specificity of 85.0%. In our proof-of-concept study, we observed that the Raman system has a higher diagnostic accuracy when compared to CA125 especially in early stage EOCs. The Hp detection process takes < 10 min to complete. Hence, our custom-built Raman system could potentially be used as an intraoperative diagnosis setup to rapidly distinguish between benign and malignant ovarian cysts in the operating theatres.

## Data Availability

Data generated or analyzed during this study are included in the main article or supporting information.
